# Text Mining of the Classical Medical Literature for Medicines That Show Potential in Diabetic Nephropathy

**DOI:** 10.1155/2014/189125

**Published:** 2014-03-13

**Authors:** Lei Zhang, Yin Li, Xinfeng Guo, Brian H. May, Charlie C. L. Xue, Lihong Yang, Xusheng Liu

**Affiliations:** ^1^Nephropathy Department, Guangdong Provincial Hospital of Chinese Medicine, 111 Dade Road, Guangzhou 510120, China; ^2^Guangzhou University of Chinese Medicine, Guangzhou 510405, China; ^3^Evidence-Based Medicine and Clinical Research Service Group, Guangdong Provincial Hospital of Chinese Medicine, 111 Dade Road, Guangzhou 510120, China; ^4^Traditional and Complementary Medicine Research Program, Health Innovations Research Institute, WHO Collaborating Centre for Traditional Medicine, School of Health Sciences, RMIT University, Bundoora, VIC 3083, Australia

## Abstract

*Objectives*. To apply modern text-mining methods to identify candidate herbs and formulae for the treatment of diabetic nephropathy. *Methods*. The method we developed includes three steps: (1) identification of candidate ancient terms; (2) systemic search and assessment of medical records written in classical Chinese; (3) preliminary evaluation of the effect and safety of candidates. *Results*. Ancient terms Xia Xiao, Shen Xiao, and Xiao Shen were determined as the most likely to correspond with diabetic nephropathy and used in text mining. A total of 80 Chinese formulae for treating conditions congruent with diabetic nephropathy recorded in medical books from Tang Dynasty to Qing Dynasty were collected. Sao si tang (also called Reeling Silk Decoction) was chosen to show the process of preliminary evaluation of the candidates. It had promising potential for development as new agent for the treatment of diabetic nephropathy. However, further investigations about the safety to patients with renal insufficiency are still needed. *Conclusions*. The methods developed in this study offer a targeted approach to identifying traditional herbs and/or formulae as candidates for further investigation in the search for new drugs for modern disease. However, more effort is still required to improve our techniques, especially with regard to compound formulae.

## 1. Introduction

Natural products used in traditional medicine have historically been invaluable for drug development [1, 2]. Successful examples of transformation of traditional medicines into modern drugs included quinine [3], huperzine [4], aspirin [5], and artemisinin [6, 7]. However, the path from traditional medicine to pharmaceutical product is fraught with challenges. The first step is “discovery” from traditional medicine [8]. Traditional Chinese medicine, which has been “clinically” tested for thousands of years, is a rich source of therapeutic leads for drug discovery. These ancient remedies were handed down from generation to generation and recorded in the classical literatures. Nowadays, the classical medical books have become the precious cultural heritage in China, and they are important sources for drug discovery from traditional medicine. As researchers in Western countries have focused on translational medicine to develop more effective clinical strategies from laboratory results, scholars in China have begun to search for potentially effective natural products based on these historical records of medical experience [8–10].

However, as the years passed, diseases and their names changed, leading to the disassociation between the traditional and modern medical terminologies. Given the voluminous content of the traditional Chinese medical literature, conducting searches to identify potential drug candidates is challenging. Additionally, the effects of classical formulae for the treatment of modern diseases still need to be assessed. All of these aspects present obstacles to the effective and efficient use of the classical literature resources for therapeutic product discovery. Consequently, modern approaches that can mine these classical medical records of traditional Chinese medicine need to develop. Over the last five years, through the International Research Network for Traditional and Complementary Medicine (IRN-TCM), we have developed and refined methods for text mining of the traditional Chinese medicine classical literature to identify candidate herbs and herbal combinations that show potential for further research [11–13].

Diabetic nephropathy is the most common cause of end-stage renal disease around the world and is characterized by rapid progression and a poor prognosis [14]. With the standard therapy of angiotensin-converting enzyme (ACE) inhibitors or angiotensin II receptor blockers (ARB), combined with glucose, lipid, and blood pressure control [15], the outcome for patients with diabetic nephropathy remains poor [16]. There is a need for new therapies to improve the outcomes of diabetic nephropathy treatment. In China, after thousands of years of traditional medical practice, a great deal of valuable experience has accumulated regarding diabetic nephropathy. Therefore this study aimed to apply modern text-mining methods to identify candidate herbs and formulae for the treatment of diabetic nephropathy.

The project involved three parts: (1) identification of classical terms that could refer to diabetic nephropathy; (2) text mining of the classical Chinese medical literature; and (3) preliminary evaluation of the effect and safety of candidates on diabetic nephropathy and the selection of candidates for further drug discovery efforts.

## 2. Methods 

In order to identify all the classical terms that could have referred to diabetic nephropathy, literature searches were conducted. Articles that focused on original researches related to classical medical terms and on the experience of venerable TCM doctors were retrieved from the Chinese databases CNKI, VIP, Wan Fang, CBM, and TCM online. Medical textbooks for undergraduate and postgraduate teaching issued by the state and medical monographs on diabetic nephropathy were also collected through the library of Guangzhou University of Chinese Medicine.

Two authors extracted the classical terms related to diabetic nephropathy that were mentioned in these sources and calculated the frequency of mention for each term. In order to obtain expert opinion on which terms were more corresponding with diabetic nephropathy, a questionnaire was designed and distributed to traditional medicine hospitals around China. Heads of the nephrology department in these hospitals who had more than 10 years of clinical experience in classical medical Chinese were consulted.

The consulting questionnaire included all the classical terms, classical medical records describing their clinical manifestations, and the clinical features of diabetic nephropathy according to the diagnostic criteria of modern medicine. Experts were required to gauge the degree of consistency between the classical term and the modern conception of diabetic nephropathy by comparing their clinical manifestations descriptions. Frequencies of each classical term mentioned in research articles, empirical articles, textbooks, and medical monographs were attached as a reference.

The degree of consistency was divided into five categories: completely consistent (5 points), mostly consistent (4 points), partly consistent (3 points), seldom consistent (2 points), and completely inconsistent (1 point). Experts had to tick only one category for each classical term. Total score of each classical term was calculated by adding the points experts ticked. Scoring rate of each classical term was full score divided by its total score and then multiplied by 100%. Full score was 5 points multiplied by the number of returned questionnaires.

These classical terms with scoring rate more than 50 percent were regarded as identified terms by expert consultation for further verification. Their corresponding modern diseases were retrieved in the textbooks and monographs of Chinese Internal Medicine, monographs of kidney disease of Chinese Medicine, and dictionaries of Chinese Medicine via the library of Guangzhou University of Chinese Medicine. The mentioned frequencies of each modern disease were counted.

Classical terms which have corresponding modern diseases not limited to diabetic nephropathy or targeting many organs not mainly in kidney were excluded. Classical terms with corresponding modern diseases which refer to kidney damages occurring in diabetes mellitus were included and used in ancient literature searching.

“Encyclopedia of Traditional Chinese Medicine” (CD-ROM version 4.0, published by Hunan Electronic and Audio-Visual Publishing House in 2006), which includes 1009 different Chinese medical books written before the emergence of the People's Republic of China (1949 AD) [13], was selected as the text mining resource.

The information about the treatments of these included classical terms was extracted, including the titles and completion dates of the books, all records related to therapies for disorders congruent with diabetic nephropathy, and the formulae used for treating these disorders. Ancient formulae targeting incongruent disorders with diabetic nephropathy confirmed by two authors, respectively, were excluded. Discrepancies were resolved by a third author, who made the final decision. The frequency of citation of each included formula was calculated. Formulae with higher recorded frequency were selected as candidates for further work in drug discovery for diabetic nephropathy.

A preliminary evaluation of the effect of candidates on diabetic nephropathy was conducted by searching the databases PubMed (January 1966 to June 2012), EMBASE (January 1985 to June 2012), the Cochrane Library, and clinicalTrials.gov to locate studies on the clinical application and experimental research on candidate formulae and their components.

## 3. Results

### 3.1. Classical Terms That Refer to Diabetic Nephropathy

Database searches resulted in the inclusion of 91 original research articles and 25 articles on the empirical experience of venerable TCM doctors that mentioned classical terms for DN. 60 medical monographs and 11 textbooks that included sections on diabetic nephropathy were selected via the library of Guangzhou University of Chinese Medicine.

A total of 31 classical terms associated with diabetic nephropathy were collected for expert consultation (Table 1). Frequencies of each classical term mentioned in research articles, empirical articles, medical monographs, and textbooks were attached as a reference (Table 1).

Thirty-five questionnaires were returned from 4 municipalities, 17 provinces, and 3 autonomous regions in China. These did not include Shandong province, Hainan province, Gansu province, Hunan province, Qinghai province, Tibet autonomous region, and the Xinjiang Uygur autonomous region. Full score of each classical term was 175 points (5 points multiplied by 35 returned questionnaires). Scoring rates of Shui Zhong (*水肿*), Shen Xiao (*肾消*), Niao Zhuo (*尿浊*), Guan Ge (*关格*), Xu Lao (*虚劳*), Xia Xiao (*下消*), Xiao Ke (*消渴*), and Xiao Shen (*消肾*) were more than 50 percent (Table 2). Experts who marked one classical term at least 3 points were considered approving the consistency between this classical term and diabetic nephropathy and their provinces were listed in Table 2.

To further verify the consistency between classical terms with scoring rate more than 50 percent and diabetic nephropathy, 35 textbooks of Chinese Internal Medicine, 86 monographs of Chinese Internal Medicine, 57 monographs of kidney disease of Chinese Medicine, and 12 dictionaries of Chinese Medicine were retrieved via the library of Guangzhou University of Chinese Medicine. The correspondence between these ancient terms and diabetic nephropathy was overlapping (Table 3).

Corresponding modern diseases of Shui Zhong (*水肿*) include renal edema, cardiac edema, nutritional edema, endocrine edema, hepatic edema, and edema of unknown reason. Besides diabetic nephropathy, renal edema also refers to acute or chronic glomerulonephritis, nephrotic syndrome, other secondary glomerular diseases (such as lupus nephritis), and chronic renal failure. Xu Lao (*虚劳*) is considered as chronic and consumptive disease involving multisystems and multiorgans, especially organ function decline or failure. Guan Ge (*关格*) is regarded as chronic renal failure, acute renal failure, uremia period, ileus, and esophageal carcinoma. Niao Zhuo (*尿浊*) refers to chyluria, phosphaturia, filariasis, urinary system infection, urinary system cancer, tuberculosis, and so on. Xiao Ke (*消渴*) mainly refers to diabetes mellitus (Table 3).

Shen Xiao (*肾消*), Xia Xiao (*下消*), and Xiao Shen (*消肾*) were not regarded as independent diseases in textbooks and monographs of Chinese Internal Medicine, and monographs of kidney disease of Chinese Medicine. They were mentioned in Xiao Ke (*消渴*) when kidney damage occurs (Table 3).

The following three extracts are examples of descriptions consistent with DN [17]. In relation to Xiao Shen (*消肾*) the *Bei Ji Qian Jin Yao Fang*, written by Sun Si-miao during the Tang Dynasty (652 AD), provides the following description: “*Patients with symptoms such as fever due to deficiency, thirst but not drinking more water, frequent urination, turbid urine and thready pulse were often diagnosed as Xiao Shen (*消肾*) disease*.” In the *Jing Yue Quan Shu*, written by Zhang Jie-bin during the Ming Dynasty (1640 AD), the following definition is provided: “*Xia Xiao (*下消*) with the symptoms of dark urine, turbid urine, gloomy complexion, muscle wasting, is also called Shen Xiao (*肾消*), as the disease location is in the kidney (Shen equals to kidney in Chinese)*.” The *Cheng Fang Qie Yong*, which was written by Wu Yi-luo during the Qing Dynasty (1761 AD), provided the following linkage with diabetes: “*Shen Xiao (*肾消*) progresses from Xiao Ke (*消渴*), with the symptoms of polydipsia, polyuria and turbid urine*.”

The relationship between the three ancient terms was described in 8 dictionaries of Chinese Medicine. Xia Xiao (*下消*) refers to Shen Xiao (*肾消*) and Xiao Shen (*消肾*).

Xiao Shen (*消肾*) refers to Xia Xiao (*下消*) and Shen Xiao (*肾消*). But besides Xia Xiao (*下消*), Shen Xiao (*肾消*) also refers to Qiang Zhong (*强中*), which is called priapism in modern times. Therefore, Xia Xiao (*下消*), Shen Xiao (*肾消*), and Xiao Shen (*消肾*) were considered more corresponding with diabetic nephropathy. However, ancient records about the symptoms of priapism should be excluded during the ancient formulae information extraction.

### 3.2. Discovery from the Classical Medical Literature Text Mining

This study searched ancient records of Xia Xiao (*下消*), Shen Xiao (*肾消*), and Xiao Shen (*消肾*) via “Encyclopedia of Traditional Chinese Medicine.” Ancient records which were thought to be corresponding with the symptoms of priapism were not included for formulae extraction.

The search revealed a total of 80 Chinese formulae for treating disorders congruent with diabetic nephropathy recorded in medical books from Tang Dynasty (618 AD to 907 AD) to Qing Dynasty (1644 AD to 1912 AD). The earliest formulae for treating diabetic nephropathy recorded in Tang dynasty were Huang qi yin (*黄芪饮*), Xuan bu wan (*宣补丸*), and E jiao tang (*阿胶汤*). Eighteen formulae were recorded more than 5 times. The top eight formulae were, in the order, Liu wei di huang wan (*六味地黄丸*), Jia jian shen qi wan (*加味肾气丸*), Bai fu ling wan (*白茯苓丸*), Si wu tang (*四物汤*), Sao si tang (*缫丝汤*), Hui xiang san (*茴香散*), Lu rong wan (*鹿茸丸*), and Ren shen san (*人参散*) (Table 4).

The number of ingredients of the eighteen most frequent formulae was calculated in order to identify simple formulae that may be suitable for further drug discovery efforts (Table 5). The following formulae contained fewer than 5 ingredients: Sao si tang (*缫丝汤*), Hui xiang san (*茴香散*), Si wu tang (*四物汤*), Ge gen wan fang (*葛根丸*), and Gu ben wan (*固本丸*). Liu wei di huang wan (*六味地黄丸*), Jia jian shen qi wan (*加减肾气丸*), Lu rong wan (*鹿茸丸*), Hu fen san (*胡粉散*), and E jiao tang (*阿胶汤*) contain more than 5 and fewer than 10 ingredients. Bai fu ling wan (*白茯苓丸*), Shen li san (*肾沥散*), Xuan bu wan fang (*宣补丸* *方*), Cong rong wan (*苁蓉丸*), Shuang bu wan (*双补丸*), Gou qi zi wan (*枸杞子丸*), and Ping bu wan (*平补丸*) contained more than 10 ingredients (Table 5).

### 3.3. Preliminary Evaluation of the Effect and Safety of Candidates on Diabetic Nephropathy

After identification of the candidate formulae, preliminary evaluation of their effect on diabetic nephropathy was undertaken. This began with the simple, high frequency formulae. Among the 18 formulae, “Sao si tang (*缫丝汤*) (also called Reeling Silk Decoction)” ranked fifth and was the simplest since it only contained one ingredient—silkworm and/or silk cocoon.

The earliest record of its use was in *Yi Xue Zheng Zhuan *written by Yu Tuan during the Ming Dynasty (1515 AD). In reference to the inherited formula Reeling Silk Decoction, he wrote that “*it has an excellent effect on Shen Xiao with the symptoms of turbid urine, polydipsia and excessive appetite but the person loses weight*….*. the effect of the hot water used in reeling silk (i.e. Reeling Silk Decoction) is best. If this is not available, it can be replaced by a decoction of silkworm cocoon or silk floss*.”—from* Yi Xue Zheng Zhuan *[17].

Based on this report and subsequent repeated citation of this remedy by other authors, we conducted the literature search of the modern studies regarding the silkworm, its related products, and its active ingredients, for treating diabetic nephropathy in order to investigate whether this simple formula could have the potential to be developed into a new agent for diabetic nephropathy.

**Figure 1:**
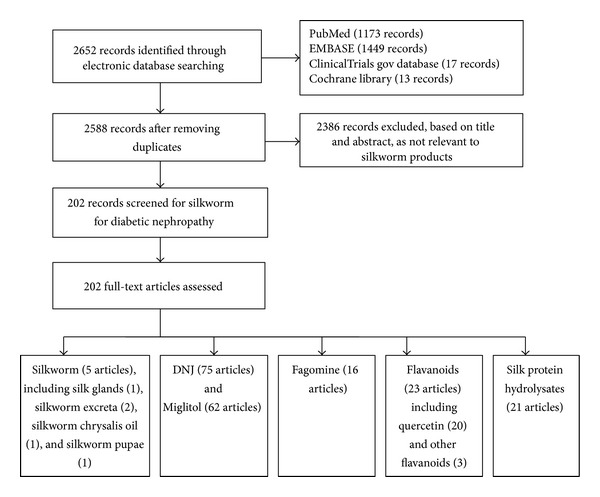
Flowchart detailing study selection process.

No studies of Reeling Silk Decoction were located, but there have been considerable studies involving silkworm, its related products, and its active ingredients. 202 articles describing the active ingredients of the silkworm and its products for diabetic nephropathy were retrieved in a search of the modern literature (Figure 1).

According to modern studies, the silkworm and its products are rich in various active substances such as alkaloids, flavanoids, and silk protein hydrolysates.

1-Deoxynojirimycin (DNJ) is a major component of the alkaloids in silkworm [18]. A clinical study in Japan [19] showed that the N-hydroxyethyl derivative of 1-DNJ (miglitol) decreased the urinary albumin excretion rate in Japanese patients with type 2 diabetes. One possible mechanism is related to improved insulin resistance [20]. It was reported to be safe for patients with stage 3 diabetic nephropathy [21]. However, it is not recommended for patients with renal insufficiency (serum creatinine >2 mg/dL) because it is excreted primarily via the kidney [22].

Among the flavanoids, which have been purified and identified from the sericin layer of silkworm cocoons [23], quercetin was reported to have renal protective effects. It suppressed glomerular mesangial cell hypertrophy, proliferation, and extracellular matrix accumulation, all of which occur in glomerular sclerosis [24]. Proposed mechanisms of action include inhibition of transforming growth factor-*β*1 (TGF-*β*1) expression [25] and amelioration of oxidative stress [26], which have been shown to be final common mediators of renal injury in diabetes [27]. Additionally, quercetin was reported to reduce nuclear factor-*κ*B (NF-*κ*B) expression, which may be involved in the pathogenesis of proteinuria in diabetic nephropathy [28, 29].

Additionally, the concentrations of 1-DNJ and the activities of quercetin in silkworm are higher than in mulberry leaves, which are the only food source of silkworm, because of the biotransformation in the silkworm body [30–35].

Therefore, components of Reeling Silk Decoction have demonstrated promising potential for development as new agents for the treatment of diabetic nephropathy. However, its safety for patients with renal insufficiency should be evaluated in further investigations.

## 4. Discussion

The methods used in classical traditional Chinese medicine, which have been “clinically” tested for thousands of years, continue to play an indispensable role in the treatment of chronic diseases in Asian countries. It has also become an important source of drug discovery for Western scholars and pharmacologists. However, barriers such as the disassociation between the traditional and modern medical terminologies, and the voluminous content of traditional Chinese medical literature, have slowed the pace of drug discoverer using the resources of the classical literature. The use of modern technology and methods for text mining of the traditional Chinese medicine classical literature can provide an approach to accelerating this process.

According to the method we developed, the process of drug discovery from the classical medical literature includes three main steps: (1) identification of candidate classical terms; (2) systemic search and analysis of classical medical records; (3) preliminary evaluation of the effects and safety of the candidates.

The usual method for identifying ancient terms corresponding with modern disease is based mainly on narrative reviews of the classical literature. However, the result in this study indicated that correspondence between ancient terms and modern disease was overlapping, rather than there being a one-to-one correspondence. This phenomenon also appeared in age-related dementia and memory impairment [12]. So the usual approach narrative review was not enough to identify the classical terms of modern disease. The two-way confirmation of terminology correspondence was applied in our study. Expert consultation was used to identify the ancient terms related to diabetic nephropathy. And then the corresponding modern diseases of each term identified by expert opinion were retrieved in textbooks and monographs of Chinese Internal Medicine, monographs of kidney disease of Chinese Medicine, and dictionaries of Chinese Medicine.

Among these identified ancient terms, Shui Zhong (*水肿*) was named after symptom of a visible edema caused by discords in many systems. Besides diabetic nephropathy, chronic or acute glomerulonephritis, nephrotic syndrome, and other secondary glomerular diseases may result in renal edema as well, which is usually characterized by facial or lower limb swelling due to water-sodium retention or hypoproteinemia. Chronic renal failure was one of the modern diseases corresponding with Guan Ge (*关格*) and Xu Lao (*虚劳*). It was the serious end stage of all the progressed chronic kidney diseases, not only diabetic nephropathy. Xiao Ke (*消渴*) was regarded as diabetes mellitus, which referred more to diabetes without kidney damage. And the modern diseases of Niao Zhuo (*尿浊*) would prefer chyluria, tuberculosis, urinary system infection, and cancer, rather than diabetic nephropathy. Therefore, it was difficult to identify that if the ancient literature describing these classical terms referred to diabetic nephropathy or not. It deserved further researches specifically identifying treatment related to diabetic nephropathy in their ancient records for each of them. Xia Xiao (*下消*), Shen Xiao (*肾消*), and Xiao Shen (*消肾*) which meant kidney damage occurring in diabetes were considered more corresponding with diabetic nephropathy and used in ancient literature text mining. Because Shen Xiao (*肾消*) also referred to Qiang Zhong (*强中*), which meant priapism in modern times. Formulae targeted Qiang Zhong (*强中*) was excluded when formulae extracting.

The two-way confirmation of terminology correspondence showed the overlap between ancient terms and modern disease more clearly. It was helpful for consistency evaluation between classical text that described these ancient terms and diabetic nephropathy in ancient text mining. However, it would be more convincing if expert consultation was included in modern diseases retrieval, just as done in the classical terms identification of diabetic nephropathy.

The systematic search of full texts of medical book firstly required the identification of a suitable collection. Our previous work located fourteen collections of traditional Chinese medical literature that could be used as resources for systematic searches [36]. The most accessible of the large full-text collections is the Zhong Hua Yi Dian CD (“Encyclopedia of Traditional Chinese Medicine”), which allows electronic searches. So the Zhong Hua Yi Dian CD was used in our study.

Since reports about the nephrotoxicity of Chinese Medicine appeared in 1994, and a condition named “Chinese herbs nephropathy” [37] received attention, the effect and safety of Chinese Medicine on patients with chronic kidney disease have been constantly questioned. Therefore a preliminary evaluation of the effect and safety of a formula is an essential step in the drug discovery process. In this study, the primary evaluation was in the form of a review of the modern literature. This provided much useful data which had some implications for further clinical investigations and pharmacology and pharmacodynamics experiments. For example, the review indicated that the active ingredients of silkworm, such as 1-deoxynojirimycin (DNJ) and quercetin, may have a renoprotective function, but this still needs further clinical verification with a large sample and in-depth molecular mechanism research. We also learnt that the safety of silkworm in diabetic nephropathy patients with renal insufficiency had to be evaluated in further investigations because of the renal excretion of 1-DNJ.

We chose Reeling Silk Decoction, which contains only a single agent, as an example in this study, since most researchers pay more attention to individual agents than to compound formulae. This is because a single agent is simple and its effect on modern disease is easier to be elucidated using current technology. However, formulae consisting of only a single agent are not typical of the prescription used in ancient China. In fact, the compound formula containing multiple agents with different roles in treating the diseases is the essence and characteristic feature of traditional Chinese medicine [9]. In our study, a total of 80 classical formulae for treating conditions congruent with diabetic nephropathy were collected. Most of these formulae are multiherb formulae, comprising two or more herbs. If researchers only focus on single agent, it is likely that they would lose much useful information. However challenges such as the unpredictable pharmacokinetic properties of multiple components and the potential risks of agent-agent interactions in formula add to the difficulty in undertaking a preliminary evaluation of formula effect and safety. More effort is still needed to improve our modern techniques in the preliminary evaluation on the effect and safety of candidates.

## 5. Conclusions

This convergence of the results of text mining of the classical literature and searches of modern biomedical databases illustrates the value of this text-based approach to the selection of candidates for drug discovery endeavours. The use of modern technology for text mining the classical literature of traditional Chinese medicine shows potential and could be an important step towards a brighter future for drug discovery. The methods developed in this study offer a targeted approach to identifying traditional herbs and/or formulae as candidates for further investigation in the search for new drugs for modern diseases. However, more effort is still required to improve our techniques, especially with regard to compound formulae.

## Figures and Tables

**Table 1:** Candidate classical terms related to diabetic nephropathy.

Classical term (Chinese name)	Frequency		
	Modern original research articles	Empirical articles by venerable TCM doctors	Medical monographs and textbooks
Shui Zhong (*水肿*)	45	16	24
Guan Ge (*关格*)	39	3	17
Niao Zhuo (*尿浊*)	35	9	5
Shen Xiao (*肾消*)	29	3	3
Xiao Ke (*消渴*)	28	12	0
Xiao Shen (*消肾*)	17	1	0
Xu Lao (*虚劳*)	17	10	14
Shen Lao (*肾劳*)	17	0	2
Xia Xiao (*下消*)	13	2	0
Yao Tong (*腰痛*)	11	3	0
Zhang Man (*胀满*)	10	1	3
Long Bi (*癃闭*)	6	0	0
Xiao Dan (*消瘅*)	5	0	1
Ni Du (*溺毒*)	5	0	0
Shui Bing (*水病*)	4	0	0
Tu Ni (*吐逆*)	4	0	0
Nei Xiao (*内消*)	3	0	0
Xuan Yun (*眩晕*)	2	3	6
San Xiao (*三消*)	2	0	0
Shen Feng (*肾风*)	1	0	0
Fei Xiao (*肺消*)	1	0	0
Shen Ke (*肾渴*)	1	0	0
Shen Shui (*肾水*)	1	0	0
Shen Dan (*肾瘅*)	1	0	0
Shen Zhuo (*肾着*)	1	0	0
Shen Jue (*肾绝*)	1	0	0
Lao Lin (*劳淋*)	1	0	0
Lin Zheng (*淋证*)	1	0	0
Xue Niao (*血尿*)	1	0	0
Pi Dan (*脾瘅*)	1	0	0
Shui Qi Bing (*水气病*)	1	0	0

**Table 2:** Results of the expert consultation.

Classical term (Chinese name)	Total score	Scoring rate (full score divided by total score)	Provinces of experts who chose completely consistent, mostly consistent, or partly consistent
Shui Zhong (*水肿*)	124	71.4%	Beijing, Guangdong, Guangxi, Guizhou, Hebei, Heilongjiang, Hubei, Jilin, Jiangsu, Jiangxi, Liaoning, Ningxia, Shanxi, Shaanxi, Shanghai, Tianjin, Yunnan, Zhejiang, Chongqing
Guan Ge (*关格*)	92	52.6%	Beijing, Guangdong, Guangxi, Guizhou, Hebei, Heilongjiang, Hubei, Jilin, Jiangxi, Liaoning, Ningxia, Shanghai, Chongqing
Niao Zhuo (*尿浊*)	95	54.3%	Beijing, Guangdong, Hebei, Heilongjiang, Hubei, Jilin, Jiangxi, Liaoning, Shanxi, Shanghai, Zhejiang
Shen Xiao (*肾消*)	111	63.4%	Beijing, Guangdong, Guangxi, Hebei, Heilongjiang, Hubei, Jiangsu, Jiangxi, Liaoning, Shanghai, Tianjin, Yunnan, Zhejiang
Xiao Ke (*消渴*)	90	51.4%	Beijing, Guangdong, Guangxi, Hebei, Heilongjiang, Hubei, Jilin, Jiangsu, Ningxia, Shanghai, Tianjin, Zhejiang
Xiao Shen (*消肾*)	88	50.2%	Beijing, Guangdong, Guangxi, Hebei, Heilongjiang, Hubei, Jilin, Liaoning, Ningxia, Shaanxi, Tianjin, Zhejiang
Xu Lao (*虚劳*)	91	52.0%	Beijing, Guangdong, Guangxi, Hebei, Heilongjiang, Hubei, Jiangxi, Liaoning, Ningxia, Shanxi, Shaanxi, Shanghai, Tianjin, Yunnan, Zhejiang
Xia Xiao (*下消*)	95	54.3%	Beijing, Guangdong, Hebei, Heilongjiang, Hubei, Jilin, Ningxia, Shaanxi, Shanghai, Tianjin, Yunnan
Shen Lao (*肾劳*)	85	48.5%	Guangdong, Guangxi, Hubei, Jilin, Jiangsu, Liaoning, Ningxia, Shanxi, Tianjin
Yao Tong (*腰痛*)	78	44.6%	Beijing, Guangdong, Hebei, Heilongjiang, Hubei, Jilin, Liaoning, Ningxia, Shanxi, Yunnan
Zhang Man (*胀满*)	63	36.0%	Beijing, Guangdong, Hebei, Heilongjiang, Hubei, Ningxia, Yunnan
Long Bi (*癃闭*)	70	40.0%	Beijing, Guangdong, Heilongjiang, Hubei, Liaoning, Shanghai, Chongqing
Xiao Dan (*消瘅*)	74	42.3%	Guangdong, Hebei, Hubei, Ningxia, Shanxi, Shaanxi, Shanghai, Tianjin, Zhejiang
Ni Du (*溺毒*)	83	47.4%	Beijing, Guangdong, Guizhou, Hebei, Hubei, Shanxi, Shanghai, Zhejiang
Shui Bing (*水病*)	84	48.0%	Beijing, Guangdong, Hebei, Heilongjiang, Hubei, Jilin, Liaoning, Ningxia, Shanxi, Shaanxi, Shanghai, Yunnan, Zhejiang, Chongqing
Tu Ni (*吐逆*)	59	33.7%	Guangdong, Guangxi, Shanxi, Shanghai
Nei Xiao (*内消*)	58	33.1%	Guangdong, Shanxi
Xuan Yun (*眩晕*)	56	32.0%	Guangdong, Hebei
San Xiao (*三消*)	68	38.9%	Beijing, Guangdong, Hebei, Ningxia, Shaanxi, Tianjin
Shen Feng (*肾风*)	57	32.6%	Guangdong, Hebei, Shanxi, Zhejiang
Fei Xiao (*肺消*)	41	23.4%	None
Shen Ke (*肾渴*)	66	37.7%	Guangdong, Hubei, Liaoning, Ningxia, Shaanxi
Shen Shui (*肾水*)	75	42.9%	Guangdong, Heilongjiang, Hubei, Jilin, Ningxia, Shanxi, Shaanxi, Zhejiang
Shen Dan (*肾瘅*)	67	38.3%	Guangdong, Hubei, Shanxi, Shaanxi, Zhejiang
Shen Zhuo (*肾着*)	55	31.4%	Guangdong, Shanxi, Tianjin
Shen Jue (*肾绝*)	46	26.3%	Hebei
Lao Lin (*劳淋*)	45	25.7%	Hebei
Lin Zheng (*淋证*)	43	24.6%	None
Xue Niao (*血尿*)	44	25.1%	Ningxia
Pi Dan (*脾瘅*)	45	25.7%	None
Shui Qi Bing (*水气病*)	77	44.0%	Guangdong, Hebei, Hubei, Jiangsu, Liaoning, Ningxia, Shanxi, Shaanxi, Tianjin, Chongqing

**Table 3:** Corresponding modern diseases of classical terms.

Classical terms	Corresponding modern diseases	Number of modern books mentioning modern disease			
		Textbooks of Chinese Internal Medicine	Monographs of Chinese Internal Medicine	Monographs of kidney disease of Chinese Medicine	Dictionaries of Chinese Medicine
Shui Zhong (*水肿*)	Renal edema	25	42	4	3
	Acute or chronic glomerulonephritis	21	40	3	Not mentioned
	Nephrotic syndrome	18	29	3	Not mentioned
	Secondary glomerular diseases (diabetic nephropathy, lupus nephritis)	3	3	Not mentioned	Not mentioned
	Chronic renal failure	Not mentioned	1	Not mentioned	Not mentioned
	Cardiac edema	23	37	4	3
	Nutritional edema	23	32	3	3
	Edema of unknown reason	6	5	2	Not mentioned
	Endocrine edema	22	30	4	3
	Hepatic edema	2	13	3	3

Xu Lao (*虚劳*)	Severe anemia	13	17	Not mentioned	1
	Immune function disorder, deficiency, or decrease	11	11	Not mentioned	Not mentioned
	Endocrine gland dysfunction	12	11	Not mentioned	Not mentioned
	Metabolic disorders	9	12	Not mentioned	Not mentioned
	Nutrition deficiency	12	11	Not mentioned	Not mentioned
	Nerve function depression or excessive suppression	9	10	Not mentioned	Not mentioned
	Organ function decline	8	4	Not mentioned	1
	Cachexia	1	Not mentioned	Not mentioned	Not mentioned
	Cancer	Not mentioned	4	Not mentioned	1
	Renal failure	Not mentioned	5	Not mentioned	1
	Heart failure	1	4	Not mentioned	Not mentioned
	Chronic respiratory disease	Not mentioned	2	Not mentioned	1
	Digestive system disease	Not mentioned	2	Not mentioned	Not mentioned
	Connective tissue diseases	Not mentioned	1	Not mentioned	Not mentioned

Guan Ge (*关格*)	Chronic renal failure	7	15	4	Not mentioned
	Acute renal failure	2	12	4	Not mentioned
	Uremia period	1	5	Not mentioned	2
	Ileus, esophageal carcinoma	Not mentioned	1	Not mentioned	Not mentioned

Niao Zhuo (*尿浊*)	Chyluria	6	9	3	Not mentioned
	Phosphaturia	Not mentioned	8	3	Not mentioned
	Filariasis	2	2	Not mentioned	Not mentioned
	Prostatitis	2	3	Not mentioned	Not mentioned
	Prostatic hyperplasia	Not mentioned	1	Not mentioned	Not mentioned
	Vesiculitis	Not mentioned	1	Not mentioned	Not mentioned
	Urinary system infection	Not mentioned	1	3	Not mentioned
	Urinary system cancer	Not mentioned	6	2	Not mentioned
	Tuberculosis	1	6	2	Not mentioned

Xiao Ke (*消渴*)	Diabetes mellitus	22	48	2	Not mentioned

Xia Xiao (*下消*)	Kidney damage occurring in Xiao Ke (*消渴*)	17	42	2	Not mentioned

Shen Xiao (*肾消*)	Kidney damage occurring in Xiao Ke (*消渴*)	3	3	2	Not mentioned

Xiao Shen (*消肾*)	Kidney damage occurring in Xiao Ke (*消渴*)	2	1	Not mentioned	Not mentioned

**Table 4:** 80 ancient formulae for diabetic nephropathy.

Pin Yin names of ancient formulas (Chinese name)	Number of ancient formulae	Recorded frequency
Liu wei di huang wan (*六味地黄丸*)	1	44 times

Jia jian shen qi wan (*加减肾气丸*)	1	38 times

Bai fu ling wan (*白茯苓丸*)	1	17 times

Si wu tang (*四物汤*)	1	11 times

Sao si tang (*缫丝汤*), Hui xiang san (*茴香散*)	2	9 times

Lu rong wan (*鹿茸丸*)	1	8 times

Ren shen san (*人参散*)	1	7 times

Shen li san (*肾沥散*), Xuan bu wan fang (*宣补丸* *方*), Ge gen wan (*葛根丸*)	3	6 times

Cong rong wan (*苁蓉丸*), Shuang bu wan (*双补丸*), Gu ben wan (*固本丸*), Hu fen san (*胡粉散*), Gou qi zi wan (*枸杞子丸*), E jiao tang (*阿胶汤*), Ping bu wan (*平补丸*)	7	5 times

Xiao tu si zi wan (*小菟丝子丸*), Da bu yuan jian (*大补元煎*), You gui yin (*右归饮*), Gui pi tang (*归脾汤*), Huang lian wan fang (*黄连丸方*), Huang qi wan (*黄芪丸*)	6	4 times

Qing xin lian zi yin (*清心莲子饮*), Huang qi yin (*黄芪饮*), Ji long tang (*荠茏汤*), Jin ying bo wan (*金银箔丸*), Zhi bai ba wei wan (*知柏八味丸*), Mi yuan jian (*秘元煎*), Zuo gui yin (*左归饮*), Hua cong rong wan (*花* *苁蓉丸*), Ying long tang (*引龙汤*), Fu tu wan (*茯菟丸*), Shu yu wan (*薯蓣丸*), Shu gan di huang san (*熟干地黄散*), Tu si zi san (*菟丝子散*), Bu ying wan (*补阴丸*)	14	3 times

Yuan tu wan (*元菟丸*), Gu ying jian (*固阴煎*), Ren shen fu ling wan (*人参茯苓丸*), Bu shen di huang yuan (*补肾地黄元*), Shen xiao san (*神效散*), Zhu long san (*竹笼散*), Ge fen wan (*葛粉丸*), Gu wa tang (*古瓦汤*), Gua lou gen wan fang (*栝蒌根丸方*), Mu li wan fang (*牡蛎丸方*), Gan di huang wan (*干地黄丸*), Tie fen wan (*铁粉丸*), Sang piao xiao wan (*桑螵蛸丸方*), Shan zhu yu fang (*山茱萸方*), Ci shi yin (*磁石饮*), Ling shu tu si wan (*苓术菟丝丸*), Tian hua wan (*天花丸*), Sang bai pi tang (*桑白皮汤*), Ning fei tang (*宁沸汤*), Nv zhen tang (*女贞汤*), Hu tao wan (*胡桃丸*), Zhen zhu fen wan (*珍珠粉丸*)	22	2 times

Lin sha dan (*灵砂丹*), Xuan tu dan (*玄兔丹*), Tian wang bu xin dan (*天王补心丹*), Da bu di huang wan (*大补地黄丸*), Xia zuo yin (*下左饮*), Nei hua wan (*内化丸*), Dang gui liu huang tang (*当归六黄汤*), Di huang tang (*地黄汤*), Jin gui shun qi wan (*金匮顺气丸*), Liu shen yin (*六神饮*), Qian jin di Huang wan (*千金地黄丸*), ying su tang (*罂粟汤*), Dan sha san (*丹砂散*), Huang lian huang qi wan (*黄* *连* *黄芪丸*), Zhu yu huang qi wan (*茱* *萸* *黄芪丸*), Ren shen lu rong wan (*人* *参* *鹿茸丸*), Yuan zhi wan (*远志丸*), Tian xiong san (*天雄散*), Shen di ying zi (*生地饮子*), Wu Long tang (*乌龙汤*)	20	1 time

**Table 5:** Ingredients of the eighteen formulae.

Classical formulae Pin Yin name (Chinese name)	Number of ingredients	Latin name of ingredients
Liu wei di huang wan (*六味地黄丸*)	6	Radix Rehmannia Preparata; Fructus Corni; Rhizoma Dioscoreae; Cortex Moutan Radicis; Poria; Rhizoma Alismatis

Jia wei shen qi wan (*加味肾气丸*)	10	Radix Rehmannia Preparata; Poria; Rhizoma Dioscoreae; Cortex Moutan Radicis; Fructus Corni; Rhizoma Alismatis; Radix Achyranthis Bidentatae; Semen Plantaginis; Cortex Cinnamomi; Radix Aconiti Lateralis Preparata

Bai fu ling wan (*白茯苓丸*)	11	Poria; Fructus Rubi; Rhizoma Coptidis; Radix Ginseng; Radix Trichosanthis; Radix Rehmannia Preparata; Endothelium Corneum Gigeriae Galli; Rhizoma Dioscoreae Septemlobae; Radix Scrophulariae; Herba Dendrobii; Fructus Cnidii

Si wu decoction (*四物汤*)	4	Radix Rehmannia Preparata; Radix Paeoniae Alba; Radix Angelicae Sinensis; Rhizoma Chuanxiong

Sao si tang (*缫丝汤*)	1	Bombyx Bombycis

Hui xiang san (*茴香散*)	2	Fructus Foeniculi; Fructus Toosendan

Lu rong wan (*鹿茸丸*)	7	Cornu Cervi Pantotrichum; Radix Scutellariae; Radix Ginseng; Radix Ipomoeae hungaiensis; Herba Cistanches; Endothelium Corneum Gigeriae Galli; Semen Cuscutae

Ren shen san (*人参散*)	9	Radix Ginseng; Cornu Cervi Pantotrichum; Radix Astragali; Fructus Trichosanthis; Ootheca Mantidis; Cortex Eucommiae; Endothelium Corneum Gigeriae Galli; Fructus Corni; Semen Cuscutae

Shen li san (*肾沥散*)	17	Endothelium Corneum Gigeriae Galli; Radix Polygalae; Radix Ginseng; Radix Astragali; Ootheca Mantidis; Rhizoma Alismatis; Radix Rehmannia Preparata; Cortex Cinnamomi; Radix Angelicae Sinensis; Os Draconis; Radix Glycyrrhizae; Radix Ophiopogonis; Fructus Schisandrae; Magnetitum; Poria; Rhizoma Chuanxiong; Radix Scrophulariae

Xuan bu wan (*宣补丸*)	12	Radix Astragali; Fructus Trichosanthis; Radix Ophiopogonis; Poria; Radix Ginseng; Radix Glycyrrhizae; Rhizoma Coptidis; Rhizoma Anemarrhenae; Radix Rehmannia Preparata; Gypsum Fibrosum; Herba Cistanches; Semen Cuscutae

Ge gen wan fang (*葛根丸* *方*)	4	Radix Puerariae; Fructus Trichosanthis; Plumbum Tetroxide; Radix Aconiti Lateralis Preparata

Cong rong wan (*苁蓉丸*)	20	Herba Cistanches; Radix Rehmannia Preparata; Radix Ophiopogonis; Rhizoma Alismatis; Fructus Schisandrae; Cortex Cinnamomi; Radix Morindae Officinalis; Cortex Lycii; Radix Angelicae Sinensis; Magnetitum; Radix Astragali; Radix Ginseng; Endothelium Corneum Gigeriae Galli; Halloysitum Rubrum; Semen Allii Tuberosi; Os Draconis; Radix Glycyrrhizae; Limonitum; Cortex Moutan Radicis; Ootheca Mantidis

Shuang bu wan (*双补丸*)	16	Colla Cornus Cervi; Lignum Aquilariae Resinatum; Rhizoma Alismatis; Fructus Rubi; Poria; Radix Ginseng; Fructus Chaenomelis; Semen Coicis; Radix Astragali; Radix Rehmannia Preparata; Herba Cistanches; Semen Cuscutae; Fructus Schisandrae; Herba Dendrobii; Radix Angelicae Sinensis; Moschus

Gu ben wan (*固本丸*)	5	Radix Ginseng; Radix Rehmanniae; Radix Rehmannia Preparata; Radix Asparagi; Radix Ophiopogonis

Hu fen san (*胡粉散*)	7	Plumbum tetroxide; Galenitum; Fructus Trichosanthis; Radix Glycyrrhizae; Rhizoma Alismatis; Halloysitum Rubrum; Halloysitum Rubrum

Gou qi zi wan (*枸杞子丸*)	12	Fructus Lycii; Poria; Radix Astragali; Endothelium Corneum Gigeriae Galli; Fructus Trichosanthis; Rhizoma Alismatis; Cortex Moutan Radicis; Fructus Corni; Radix Ophiopogonis; Concha Ostreae; Ootheca Mantidis; Semen Plantaginis

E jiao tang (*阿胶汤*)	7	Colla Corii Asini; Rhizoma Zingiberis; Radix Polygalae; Radix Aconiti Lateralis Preparata; Radix Ginseng; Radix Glycyrrhizae; Fructus Cannabis

Ping bu wan (*平补丸*)	11	Semen Cuscutae; Fructus Corni; Radix Angelicae Sinensis; Fructus Alpiniae Oxyphyllae; Fructus Toosendan; Radix Achyranthis Bidentatae; Semen Trigonellae; Cortex Eucommiae; Radix Morindae Officinalis; Herba Cistanches; Olibanum

## Appendix

### Search Strategies

#1 (“kidney”)
#2 (“glucose”)
#3 (“nephropathy”)
#4 (“diabetic nephropathy”)
#5 (“diabetes”)
#6 (“Bombyx”[Mesh] OR “chymotrypsin inhibitor 13 protein, Bombyx mori”[Supplementary Concept] OR “paralytic peptide, insect”[Supplementary Concept] OR “NUE protein, silkworm”[Supplementary Concept] OR “transcription factor TFIIIR, Bombyx mori”[Supplementary Concept] OR “Moths”[Mesh] OR “7,2′-dihydroxy-8-hydroxyethyl-4′-methoxyflavane-2′-O-beta-D-glucopyranoside”[Supplementary Concept] OR “sorbitol-6-phosphatase, Bombyx mori”[Supplementary Concept] OR “30Kc6 protein, Bombyx mori”[Supplementary Concept] OR “Edf1 protein, mouse”[Supplementary Concept] OR “fibroin, silkworm”[Supplementary Concept] OR “L-3,4-dihydroxyphenylalanine decarboxylase, Bombyx mori”[Supplementary Concept] OR “BMCHIR1protein, Bombyx mori”[Supplementary Concept] OR “Bmdsx protein, Bombyx mori”[Supplementary Concept] OR “samui protein, Bombyx mori”[Supplementary Concept] OR “30kP protease A protein, Bombyx mori”[Supplementary Concept] OR “ACPIP protein, Bombyx mori”[Supplementary Concept] OR “fhx protein, Bombyx mori”[Supplementary Concept] OR “cecropin CMIV protein, Bombyx mori”[Supplementary Concept] OR “ZDD4 protein, Bombyx mori”[Supplementary Concept])
#7 (“1-Deoxynojirimycin”)
#8 (“miglitol”)
#9 (“fagomine”)
#10 (“quercetin”)
#11 (“silk fibroin”)
#12 #1 OR #2 OR #3 OR #4 OR #5
#6 AND #12 482(pubmed)
#7 AND #12 385(pubmed)
#8 AND (#1 OR #3 OR #4) 4(pubmed)
#9 AND #12 9(pubmed)
#10 AND (#1 OR #3 OR #4) 271(pubmed)
#11 AND #12 22(pubmed)

## Abbreviations

ACE: Angiotensin-converting enzyme
ARB: Angiotensin II receptor blocker
DN: Diabetic nephropathy
GFR: Glomerular filtration rate
DNJ: 1-Deoxynojirimycin
TGF-*β*1: Transforming growth factor
NF-*κ*B: Nuclear factor.
